# Risk factors for severe COVID-19 differ by age for hospitalized adults

**DOI:** 10.1038/s41598-022-10344-3

**Published:** 2022-04-28

**Authors:** Sevda Molani, Patricia V. Hernandez, Ryan T. Roper, Venkata R. Duvvuri, Andrew M. Baumgartner, Jason D. Goldman, Nilüfer Ertekin-Taner, Cory C. Funk, Nathan D. Price, Noa Rappaport, Jennifer J. Hadlock

**Affiliations:** 1 Institute for Systems Biology, 401 Terry Ave N, Seattle, WA 98109 USA; 2 Washington University School of Medicine, St. Louis, MO 63110 USA; 3 Swedish Center for Research and Innovation, Seattle, WA 98109 USA; 4 Providence St. Joseph Health, Renton, WA 98057 USA; 5 Division of Allergy & Infectious Diseases, University of Washington, Seattle, WA 98109 USA; 6 Department of Neuroscience, Department of Neurology, Mayo Clinic Jacksonville, Jacksonville, FL 32224 USA; 7 Onegevity, a Division of Thorne HealthTech, New York, NY USA

**Keywords:** Machine learning, Epidemiology, Viral infection, Risk factors

## Abstract

Risk stratification for hospitalized adults with COVID-19 is essential to inform decisions about individual patients and allocation of resources. So far, risk models for severe COVID outcomes have included age but have not been optimized to best serve the needs of either older or younger adults. Models also need to be updated to reflect improvements in COVID-19 treatments. This retrospective study analyzed data from 6906 hospitalized adults with COVID-19 from a community health system across five states in the western United States. Risk models were developed to predict mechanical ventilation illness or death across one to 56 days of hospitalization, using clinical data available within the first hour after either admission with COVID-19 or a first positive SARS-CoV-2 test. For the seven-day interval, models for age ≥ 18 and < 50 years reached AUROC 0.81 (95% CI 0.71–0.91) and models for age ≥ 50 years reached AUROC 0.82 (95% CI 0.77–0.86). Models revealed differences in the statistical significance and relative predictive value of risk factors between older and younger patients including age, BMI, vital signs, and laboratory results. In addition, for hospitalized patients, sex and chronic comorbidities had lower predictive value than vital signs and laboratory results.

## Introduction

The number of global confirmed cases with severe acute respiratory syndrome Coronavirus 2 (SARS-CoV-2) infection has surpassed 257 million as of December 10, 2021, with over 5 million reported deaths^1^. Although the majority of patients infected by SARS-CoV-2 present with mild symptoms, studies reported that 20% get hospitalized and 5% of patients with Coronavirus disease 2019 (COVID-19) become critically ill^2,3^. From early on of the pandemic, both age and chronic comorbidities have been reported as a significant risk factor for poor outcomes^4,5^, and evidence supports increased risk with hypertension, diabetes, chronic obstructive pulmonary disease, chronic renal disease, and cardiovascular conditions^4,6,7^. Although young patients have a lower prevalence of comorbidities than aging patients, the relative risk of fatal outcome in young patients with hypertension, diabetes and cardiovascular diseases has been shown to be higher than in elderly patients^8,9^. In addition, some studies show the patient population tends to be younger with the emergence of delta as the variant of concern in the U.S. with regional proportions being greater than 99% as of November 2021^10^. Assessing risk for severe COVID-19 in specific age groups is complicated by both the heterogeneity of clinical presentation and age-related differences in the prevalence of chronic multimorbidities. A deeper understanding of risk factors for COVID-19 severity among different age subpopulations is needed, as well as practical, explainable risk stratification for bedside clinical decision support, research stewardship, and advancing our biomedical understanding of SARS-CoV-2.

Several studies have described successful development of machine learning models to predict COVID-19 outcomes in hospitalized patients^11–20^. Further, explainable models can also inform care decisions by showing which factors lead a specific individual patient to be at risk for severe outcomes, and can also help show which variables are most important at the population level, suggesting areas for further research investigation^21^. However, existing studies have several limitations; (1) most are based on small sample sizes from academic centers, (2) higher incidence of severe outcomes in hospitalized cohorts than are typically observed with current treatments, (3) reliance on laboratory tests that are not routinely administered to all patients, (4) lack of investigation of differences in risk factors between younger and older hospitalized patients, and (5) marginal model performance for either of age groups^13^. To address these limitations, we develop high-performing age-stratified machine-learning models to predict the severity of COVID-19 progression from 6,906 patients in community hospitals across a large geographic area in the western United States, during five months after the delta variant had become predominant and new standards of care had lowered the severe outcome incidence rate. In addition, the model was developed to require only those laboratory results that are routinely administered for all COVID-19 patients.

## Methods

### Study design and setting

This retrospective study analyzed data gathered from Providence St. Joseph Health (PSJH), a community health system with 51 hospitals and 1085 clinics across five states in the western United States: Alaska, California, Montana, Oregon, and Washington. Inclusion criteria was age ≥ 18 years and confirmation of COVID-19 by a positive PCR-based SARS-CoV-2 test result. This study was performed in compliance with the Health Insurance Portability and Accountability Act (HIPAA) Privacy Rule and was approved by the Institutional Review Board (IRB) at PSJH with Study Number STUDY2020000196 with waiver of consent. We follow STROBE reporting guidelines (Supplemental Table S6).

### Task definition

In this study, we hypothesized that age-stratified risk models for hospitalized patients with COVID-19 can accurately predict critical illness and mortality due to COVID-19 based on readily available patient data. Outcomes of patients were defined using the World Health Organization Ordinal Scale (WOS), proposed by the WHO R&D Blueprint group in their COVID-19 Therapeutic Trial Synopsis^22^**.** The WHO ordinal scale ranges from 0 (uninfected) to 8 (deceased) with gradations depending on hospitalization, supplemental oxygen, mechanical ventilation, and organ support (vasopressors, renal replacement therapy, and extracorporeal membrane oxygenation). See Supplemental Table S1. In this study, we categorized WHO ordinal scores of 3–5 as the mild cases of COVID-19 and WHO ordinal scores of 6–8 as the critical illness and death within hospitalized patients. The objective is to develop machine learning models to predict critical illness and death with COVID-19 in hospitalized patients using easily available variables, including aggregated laboratory biomarkers and vital signs within one hour of either admission to the hospital or the first positive inpatient SARS-CoV-2 test. These predictive models are developed on time horizons for one, seven, 14, 28, and 56 days from the confirmation of the infection and hospitalization to test the assumption that the baseline data up to one hour after hospital admission with a positive SARS-CoV-2 test can predict risk of critical illness on different time horizons. Additionally, we compare the performance of machine learning models within 7-days from the confirmation of the infection and hospitalization for (1) all-ages population, and (2) age-stratified subpopulations, to report the effect of age and compare the relative importance of risk factors between younger and aging adults.

### Population

The start time point of study is defined as June 31, 2021, after the delta became the predominant SARS-CoV-2 variant in the Western United States. Studied population included hospitalized individuals who received a positive test for COVID-19 between June 31, 2021 and November 15, 2021. This was confirmed by reverse-transcriptase polymerase chain reaction (RT-PCR) for the SARS-CoV-2 ribonucleic acid (RNA). Patients were excluded if they were already receiving mechanical ventilation at the time of admission to the hospital.

### Variables

The factors analyzed for prediction of COVID-19 outcomes were demographic characteristics, medical history, vital signs, and laboratory biomarkers (n = 64). We extracted the Charlson Comorbidity Index^23^ (CCI: measure of overall comorbidities) and individual chronic conditions that are known risk factors for poor COVID-19 outcomes (reported in the literature^6^) and conditions which are prevalent in aging patients (Table 1). Comorbidities that are usually chronic, such as hypertension, were included if they were active at the time of admission. Other comorbidities were included if they had been active any time within 2 years prior to admission, except for malignancy, which was included if active any time within the past 5 years. Note that active conditions mean health issues that affect the individual's current functioning and all health. We used ICD-10-CM (International Classification of Diseases, Tenth Revision, Clinical Modification) codes, which are shown together with SNOMED–CT© hierarchical parent codes (Supplemental Table S2). Laboratory results and vital signs were included (both inpatient and outpatient) if they were collected between 24 h before and one hour after either admission to the hospital or the first positive inpatient SARS-CoV-2 test (Table 1). Note that, we used aggregated temporal longitudinal vital signs in our model as described in Lee, et.al^24^. Additionally, the risk factor list included patients' need for supplemental oxygen mode, need for vasopressors, total number of comorbidities, and COVID-19 vaccination status.

**Table 1:** Demographics, vital signs, laboratory tests, and medical conditions analyzed for SARS-CoV-2 positive patients.

Demographics	Vital signs	Laboratory tests	Medical conditions	Other risk factors
Age	Heart rate (HR)	White blood cell count (WBC)	Hypertension	Initial oxygen mode
Body mass index (BMI)	Respiratory rate (RR)	Platelets (PLT)	Coronary arteriosclerosis	Total Number of comorbidities
Sex	Systolic blood pressure (SBP)	Hematocrit (HCT)	Heart failure	Vaccination status
Reported ethnicity	Diastolic blood pressure (DBP)	Hemoglobin (HGT)	Cardiomyopathy	Vasopressors
Reported race	Temperature	Basophils (BASO)	Chronic obstructive pulmonary disease (COPD)	
	Oxygen saturation (SpO_2_)	Eosinophils (EOSABS)	Asthma	
		Lymphocytes (LYMABS)	Malignancy	
		Monocytes (MONO)	Liver disease	
		Neutrophils (NEUABS)	Hyperlipidemia and Dyslipidemia	
		Potassium (K)	Obstructive sleep apnea	
		Sodium (NA)	Chronic kidney disease	
		Chloride (CI)	Diabetes mellitus	
		Bicarbonate (HCO3)	Solid organ transplant	
		Creatinine (CREA)	Conditions related to reduced immune response	
		Blood urea nitrogen (BUN)	Dementia (All Causes)	
		Glucose (GLU)		
		Albumin (ALB)		
		Alkaline (ALP)		
		Aspartate aminotransferase (AST)		
		Alanine aminotransferase (ALT)		
		Anion Gap (AGAP)		
		Bilirubin (TBIL)		
		Calcium (CA)		
		Globulin (GLOB)		
		Total Protein		
		D-dimer		
		C-reactive protein		
		Prothrombin time		
		BUN/Creatinine Ratio		
		Ferritin		
		International normalized ratio (INR)		
		Magnesium (MG)		
		Procalcitonin		
		Lactate dehydrogenase (LDH)		

### Statistical analysis

Descriptive analyses are presented as frequencies and percentage for categorical variables, and as mean and standard deviation (std) for numerical variables. Fisher exact test was applied to compare distributions of categorical variables. The differences between distributions of numerical variables were calculated using Mann Whitney U-test. All statistical analyses were completed using PySpark version 2.4.5.

### Risk model development

In data preprocessing for development of each risk model, we removed features with missing values greater than 20% (Supplemental Table S3). We used IterativeImputer from Scikit Learn version 0.24.0 for imputing missing data in numerical features^25^. Missing values for comorbidities were assumed to be absent from the patient’s medical history and imputed with a constant number of 0. Outliers were detected by calculating the modified z-score based on median absolute deviation with a threshold of 3.5 and then these outliers were imputed by the median.

To build machine learning models, we randomly split the dataset into 80% training data and 20% testing data and analyzed each patient using multiple algorithms including logistic regression (LR), random forest classification (RF), Adaptive Boosting (AdaBoost), and Gradient Boosting Decision Tree (GBDT). The parameters for each model were optimized using a tenfold cross-validation on the training set with the maximum scoring value for the area under receiver operating characteristic curve score (AUROC). We then balanced true and false positive rates by optimizing the probability threshold for each class. This optimal cut-off point is defined using the Youden index to maximize the summation of true positive rate and true negative rate.

To address collinearity between predictors, we compared the optimum performance of logistic regression using the least absolute shrinkage and selection operator (LASSO) feature selection method. For non-linear tree-based models all features were included. Performance of models was reported as the area under the receiver operating characteristic curve (AUROC), area under the precision-recall curve (AUPRC), true positive rate (TPR), true negative rate (TNR), predictive positive value (PPV), and negative predictive value (NPV). We reported the 95% confidence interval for performance metrics of the models using Wilcoxon statistics^26^, and binomial interval^27^ for the area under the ROC and precision-recall curves, respectively. All ML models were applied using Spark version 2.4.5, in the Python interface. We presented the interpretation of the model with the highest relative performance, gradient boosting, using the Shapley additive explanations (SHAP) algorithm, which uses cooperative game theory to calculate the marginal contribution of each feature, and examines the feature influence on model prediction^28^. Predictive models were reported following TRIPOD guidelines^29^.

## Results

### Baseline characteristics

In the Providence St Joseph cohort (described in “Methods” above), 6,906 patients with positive tests for SARS-CoV-2 were analyzed (Supplemental Fig. S1). The severe outcome incidence rate of 10.88%. Percent female was 44.25 and mean age was 59.90 years (SD ± 17.83 years), with a range 18 to 90 + years old. The distribution of relative frequency of hospitalizations by age is shown in Supplemental Fig. S2. We divided the patients into two age subgroups: younger (age ≥ 18 and < 50 years with 1,963 patients) and older (≥ 50 years with 4,943 patients). The reported variables for prognosis of COVID-19 critical illness are presented in Table 2, Supplemental Table S3, and Supplemental Table S4. For patients with age ≥ 18 and < 50 years, the variables that had statistically significant correlation with critical illness and death in patients with COVID-19 were BMI, age, heart failure, and cardiomyopathy. For patients with age ≥ 50 years, the statistically significant variables were BMI, age, sex, dementia, and use of vasopressors within one hour of either admission to the hospital with COVID-19 or a first positive inpatient SARS-CoV-2 test. Vital signs values were aggregated from 24 h before to one hour after and included (mean and standard deviation) for heart rate (HR), systolic blood pressure (SBP), diastolic blood pressure DBP, respiratory rate (RR), blood oxygen saturation (SpO2), and body temperature.

**Table 2:** Demographics and medical conditions among hospitalized patients with COVID-19 by severity.

Variable	Patients with age ≥ 18 and < 50 years (n = 1,963)				Patients with age ≥ 50 years (n = 4,943)			
	Mild (WOS ≤ 5) (n = 1,810)	Severe (WOS > 5) (n = 153)	P-value	OR*	Mild (WOS ≤ 5) (n = 4,349)	Severe (WOS > 5) (n = 595)	P-value	OR
Age in years, mean (std)	37.21 (8.27)	39.320 (8.15)	< 0.001	-	68.67 (11.63)	70.17 (11.64)	< 0.001	-
BMI, kg/m^2^, mean (std)	34.18 (9.46)	37.219 (1.00)	< 0.001	-	31.06 (8.31)	32.10 (9.10)	< 0.001	-
Sex (Male)	991 (54.75%)	92 (60.13%)	0.205	1.246	2367 (54.43%)	356 (59.83%)	0.014	1.247
Ethnic group (Hispanic)	565 (31.21%)	53 (34.64%)	0.415	1.168	537 (12.35%)	74 (12.44%)	0.947	1.008
Race**	742 (40.99%)	64 (41.83%)	0.864	1.035	1006 (23.13%)	152 (25.55%)	0.197	1.140
Hypertension	95 (5.25%)	6 (3.92%)	0.571	0.737	863 (19.84%)	111 (18.65%)	0.510	0.926
Coronary Arteriosclerosis	11 (0.61%)	2 (1.31%)	0.269	2.166	429 (9.86%)	50 (8.40%)	0.301	0.838
Heart failure	26 (1.44%)	6 (3.92%)	0.034	2.801	471 (10.83%)	68 (11.43%)	0.674	1.062
Cardiomyopathy	9 (0.50%)	3 (1.96%)	0.061	4.002	112 (2.57%)	13 (2.18%)	0.676	0.845
COPD	6 (0.33%)	1 (0.65%)	0.434	1.978	390 (8.97%)	46 (7.73%)	0.355	0.851
Asthma	82 (4.53%)	6 (3.92%)	1.000	0.860	226 (5.20%)	27 (4.54%)	0.552	0.867
Malignancy	39 (2.15%)	4 (2.61%)	0.573	1.219	412 (9.47%)	50 (8.40%)	0.453	0.877
Liver disease	71 (3.92%)	7 (4.57%)	0.665	1.174	232 (5.33%)	32 (5.38%)	0.923	1.009
Dyslipidemia, Hyperlipidemia	122 (6.74%)	14 (9.15%)	0.247	1.394	1188 (27.32%)	150 (25.21%)	0.302	0.897
Obstructive sleep apnea	52 (2.87%)	6 (3.92%)	0.452	1.380	347 (7.98%)	37 (6.22%)	0.142	0.765
Chronic kidney disease	27 (1.49%)	4 (2.61%)	0.298	1.773	523 (12.02%)	82 (13.78%)	0.230	1.169
Diabetes mellitus	150 (8.29%)	7 (4.57%)	0.120	0.531	756 (17.38%)	119 (20%)	0.122	1.188
Solid organ transplant	3 (0.17%)	1 (0.65%)	0.277	3.963	8 (0.18%)	3 (0.50%)	0.137	2.750
Immunosuppression	12 (0.66%)	1 (0.65%)	1.000	0.986	48 (1.10%)	7 (1.18%)	0.835	1.067
Dementia (all causes)	0 (0%)	0 (0%)	-	-	138 (3.17%)	27 (4.54%)	0.088	1.451
Vasopressors	10 (0.55%)	1 (0.65%)	0.591	1.184	16 (0.37%)	15 (2.52%)	0.000	7.004

*OR = Unadjusted odds ratio. **American Indian, Alaska Native, Asian, Black or African American, Native Hawaiian or other Pacific Islander, Other.

### Risk model analysis

**Figure 1:**
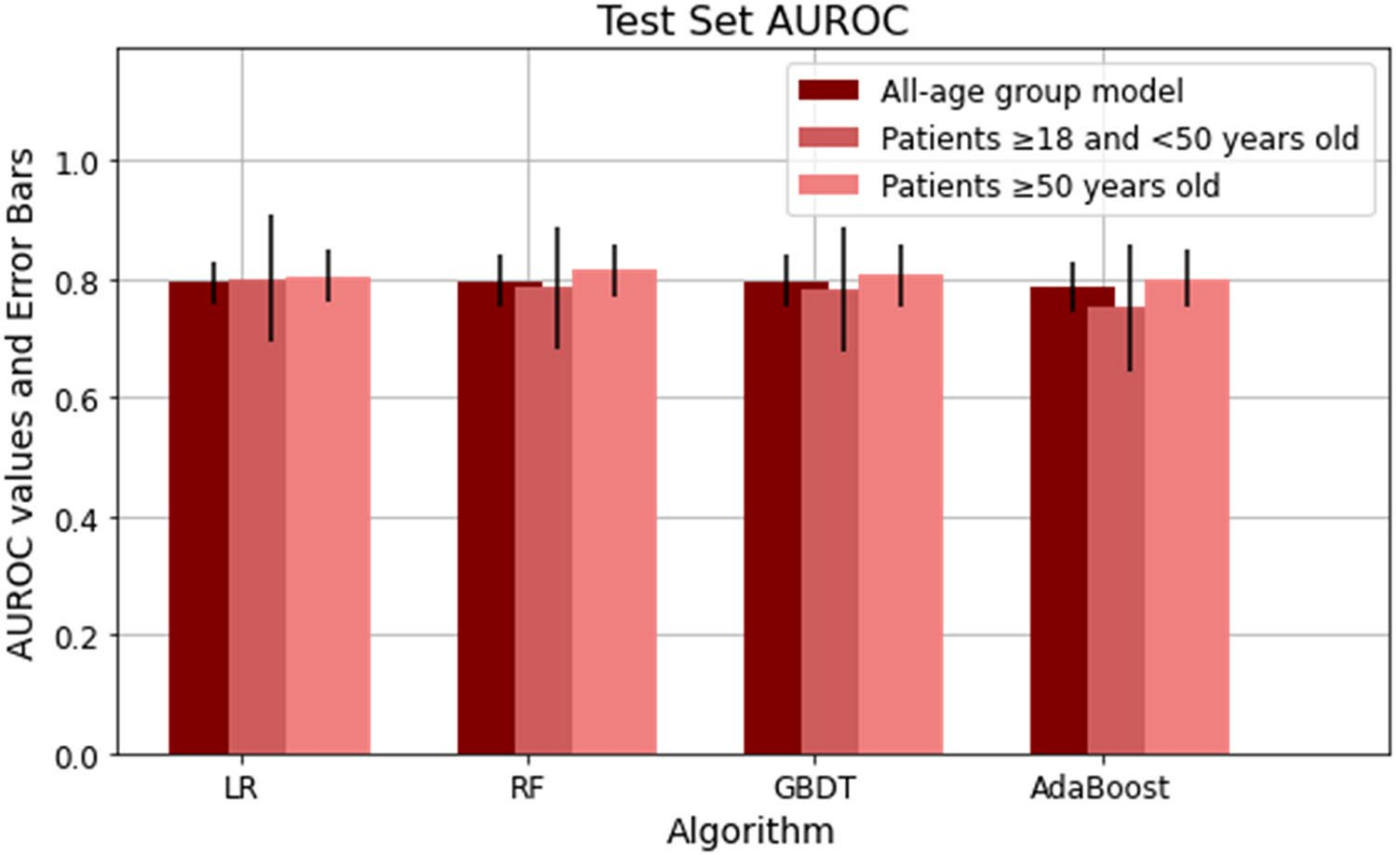
Area under receiver operator characteristic curve (AUROC) for age-stratified models of severe COVID-19 outcomes in hospitalized patients.

**Figure 2:**
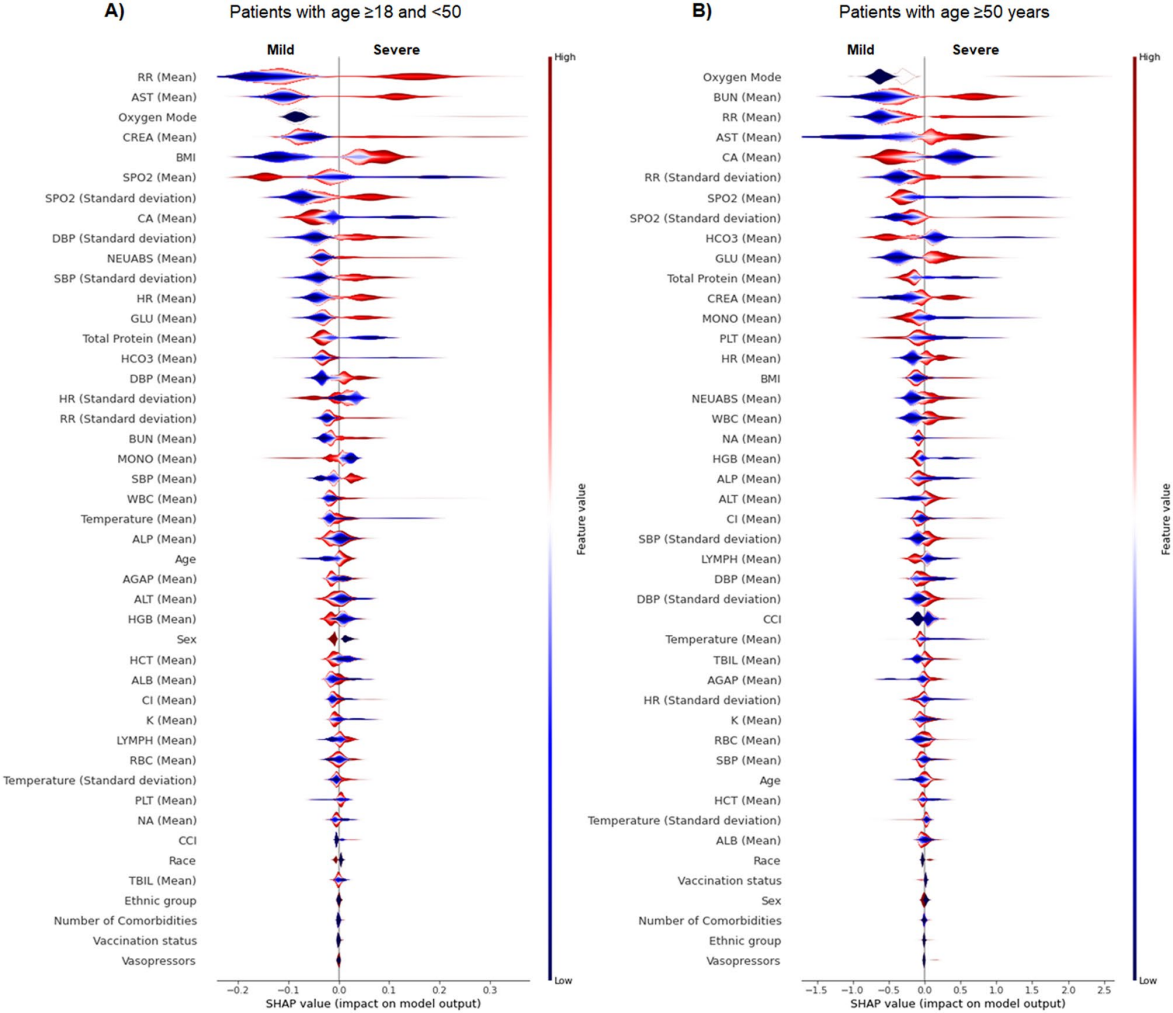
Gradient Boosting Decision Tree feature importance for age-stratified models of severe COVID-19 outcomes in hospitalized patients. (**A**) Feature importance and the influence of higher and lower values of the risk factors on the patient with age ≥ 18 and < 50 years outcome, (**B**) Feature importance and the influence of higher and lower values of the risk factors on the patient with age ≥ 50 years outcome. Note that the left side of this graph represents reduced risk of critical illness or death, and the right side of the graph represents the increased risk of critical illness and death outcome. Nominal classes are binary [0, 1]. For sex, female is 0 (blue) and for race, White is 0 (blue).

In this paper, we trained five ML models including LR, RF, GBDT, and AdaBoost for the all-age population (n = 6,906), and two different age subpopulations (patients with age ≥ 18 and < 50 years with n = 1,963 and patients with age ≥ 50 years with n = 4,943) using the aggregated values of predictors. Class distribution for outcomes show that patients with critical illness and death accounted for 7.79% of the younger cohort with age ≥ 18 and < 50 years and 12.04% of the older cohort with age ≥ 50 years. This class imbalance was addressed by undersampling patients with mild severity from the training set. Results were reported on the complete test dataset, representing actual population distribution. Supplemental Table S5 represents the performance results for three sets of developed models for younger, older patients and all-age groups. These performance results were reported after adjusting the probability threshold to optimize models for clinical and research applications. Among four models for the younger population, GBDT had the highest true positive rate of 74.98%, true negative rate of 74.04%, and AUROC value of 0.78. For the older population, GBDT had a maximum true positive rate of 72.72%, true negative rate of 72.91% and AUROC of 0.81. Figure 1 represents the comparison between the AUROC values for four ML models based on the patient's age. Relative feature importance for the younger, older and generalized GBDT models was determined by Shapley additive explanations (SHAP), as shown in Fig. 2, and Supplemental Fig. S5, respectively. SHAP values were also used to assess the contribution of age on each model outcome (Supplemental Fig. S5). In addition, Supplemental Fig. S6 presents the model with individual comorbidities as risk factors for age-stratified models. We used the distribution of importance for each variable to assess its contribution to model outcome. In the younger population, some variables for comorbidities added no predictive value, which resulted in them being automatically removed from the SHAP plot.

Additionally, we used the GBDT to validate and assess the performance of the model for different time horizons. For the all-age group, gradient boosting showed an AUROC value of 0.83, 0.80, 0.79 and 0.79 for respectively, 1-, 14-, 28- and 56-day intervals after the confirmation of infection. Furthermore, we predicted the mortality of patients (WHO ordinal score of 8) using the GBDT model and the full set of aggregated risk factors. Note that to predict the mortality of patients with COVID-19, we also included the patients who were already receiving mechanical ventilation and additional organ support (WHO ordinal score of 6 and 7), see Supplemental Fig. S2. Therefore, the number of all age group patients for predicting mortality increased to 7,063. The results show the AUROC value of 0.82 for the general population and 0.79 and 0.75 for the younger and aging population, respectively.

## Discussion

In this study, we developed risk models to predict the outcomes of hospitalized adult patients with COVID-19, in the context of current COVID-19 standard of care and delta variant predominance. We used clinical data from within one hour of either admission to the hospital with COVID-19 or the first positive inpatient SARS-CoV-2 test result. Explainability analysis on the machine learning models showed that risk factors are different for older patients compared to younger patients. This is the first study that investigates age-stratified modeling for COVID-19 severity for hospitalized adults for early prediction across multiple time horizons. Data from 6,906 patients across five states was used to develop predictive models for COVID-19 critical illness and death in younger and older hospitalized adults within one, seven, 14, 28 and 56 days of positive infection test and hospitalization. The key findings are: 1) risk models perform well using readily available clinical data, 2) vital signs and laboratory results at the time of admission are more important for prediction than the presence of comorbidities, 3) age-stratified models show that the relative importance of risk factor differs between younger and older adults.

Since the beginning of the pandemic, standard of COVID-19 care has improved and delta has become the predominant variant. Further, risk models from earlier in the pandemic relied on labs that are not routinely used in many patients. This was reflected by the high rate of missing values for tests required for in early risk scores, including INR, D-dimer, ferritin and procalcitonin (PCT). The models developed here are both performant and pragmatic.

Our statistical analysis revealed new insights on how variables that correlate significantly with critical illness and death in COVID-19 differ between younger and older age groups. For example, most comorbidities such as malignancy, cardiomyopathy and COPD have higher odds ratios for severe outcomes in younger patients than in older patients. Conversely, lower BUN/creatinine ratio and lower potassium are only statistically associated with critical illness and death in older patients.

We chose GBDT, a sequential ensemble approach^30^, as the model with the best relative performance to define the most predictive variables for COVID-19 outcomes. Non-linear models showed higher performance than linear models, suggesting better representation of complex interactions across multiple mechanisms of disease. Stratifying patients by age group revealed that, in general, vital signs and laboratory tests have a higher relative importance than comorbidities. Because age is such a significant risk factor, it can mask other important predictors. By removing the confounding effects of age, these models highlight new insights into risk factors for IMV and death.

Additionally, we investigated the effect of age on predictive models for younger and older COVID-19 patients. For patients with age ≥ 18 and < 50 years (Supplemental Fig. S5C), age has a relatively high and more consistent predictive effect on the performance of the model. Within patients younger than 50 years old, higher age had a negative effect on outcome. However, in patients with age ≥ 50 years (Supplemental Fig. S5D), age has less effect on the model performance. Patient stratification removed some of the confounding effect of age in this group, better revealing the contribution of laboratory results, vital signs and comorbidities as predictors.

For the younger population, the patient's initial oxygen mode and aggregated vital signs demonstrate the highest predictive value for outcome severity. Other predictive factors include higher AST, higher creatinine, and lower calcium levels, higher age, and higher BMI. Laboratory results have higher importance for older patients than they do for younger patients. Features such as higher BUN, higher AST, lower HCO_3_, lower calcium, and some aggregated vital signs (respiratory rate, blood pressure and SpO_2_) are among the most predictive. Sex is not a strong predictive factor, despite it having an odds ratio of ~ 1.25 in both the older and younger population. BMI is another feature that supports the importance of analyzing age subgroups separately. It is statistically correlated to the severity of COVID-19 and is an important predictor for the younger population but shows no significant correlation in the older population (Supplemental Fig. S4). This could be explained by higher BMI in younger hospitalized patients compared to the older hospitalized patients with COVID-19^31^. Future investigation is needed to determine risks with being underweight or overweight, potentially with BMI-stratified models. Neither race nor ethnicity had strong feature importance for prediction in the younger and older population. This shows that although chronic comorbidities (Charlson Comorbidity Index or binary diagnostic labels), sex, race, ethnicity may have high odds ratios in a univariate analysis, these factors are much less important in the acute setting for predicting critical illness. Once hospitalized, biomedical observations are more predictive. Chronic conditions are still important for predicting the severity of COVID-19 outcomes, but medical and clinical biomarkers have a higher predictive value. The importance of comorbidities and CCI has also been investigated by comparing a predictive model which includes only demographics and CCI. The comparison of models performance is presented in Supplementary Fig. S6 and SHAPs are presented in Supplementary Fig. S7 and Supplementary Fig. S8.

SHAP values also indicate the direction of variables’ impact on outcomes. For example, higher serum creatinine levels, lower platelet counts, lower lymphocyte counts, and higher neutrophil count are all predictive of critical illness and death^28^ Lower calcium is associated with more severe COVID-19, as noted in previous studies^32^, and this analysis shows it has higher predictive value in older patients.

Hence, age stratification shows that risk factors for severe COVID-19 differ by age, in ways that cannot be determined in all-age models. This affirms the importance of analyzing each different age group separately, particularly for the older population who have the greater overall risk for poor outcomes.

Also, as expected, vaccination reduced the risk of severe outcomes in the older population. Vaccination status had relatively low importance, which may reflect the low number of hospitalized patients who had received vaccination during the observation window; only 8.10% of the younger hospitalized patients and 25.48% of older hospitalized patients had received at least one dose of a vaccine (Supplemental Fig. S3).

Early risk stratification in patients with COVID-19 is essential to inform decisions about what level of care a patient is likely to need. One of the main challenges of COVID-19 is the heterogeneity of presentation; therefore factors related to poor outcomes are not always evident at admission^15^. In this study, ML models using readily available variables (demographics, vital signs, common laboratory test and medical history) demonstrated strong performance for predicting the severity of COVID-19. Importantly, the population in this study included patients from 51 hospitals and 1081 clinics across five states, using data based on the current standard of care for COVID-19 and the delta variant. Five limitations of this retrospective study are: 1) reliance on EHR structured data which can miss medical conditions that not diagnosed, not recorded, or noted only in free text, 2) use of hospital reported race and ethnicity of patients^33^ as opposed to direct per-patient measures of potential confounders (genetic information, disparities in healthcare, and individual lifetime history of beneficial and harmful exposures, 4) use of data from within a single healthcare system. Concerns regarding generalizability of this study are partially mitigated by the size and diversity of PSJH, which serves both urban and rural communities from California to Alaska. Future investigations will benefit from finer granularity of subdivisions by age, BMI, and more detailed variables on conditions and drugs that affect individual immune response.

## Conclusion

We developed two age-stratified risk models for critical illness in hospitalized patients with COVID-19 and tested them on data from patients during times of improved standard of care treatment and delta variant predominance. For hospitalized adults, baseline data that is readily available within one hour after hospital admission or a first positive inpatient SARS-CoV-2 test can predict critical illness within one day, and up to 56 days later. The models for age ≥ 18 and < 50 years and the model for age ≥ 50 years were both more performant than all-age models. These age-stratified models also revealed differences in the statistical significance and relative predictive value of risk factors between older and younger patients, including age, BMI, vital signs, and laboratory results. In addition, sex and chronic comorbidities had lower predictive value than vital signs and laboratory results. The results of this age-stratified modeling approach provide advanced understanding of current risk factors for severe COVID-19 outcomes and can help inform care decisions and prioritize next steps for research.

## Supplementary Information

Supplementary Information.

## Data Availability

All clinical logic has been shared within the paper and supplemental materials. Results have been aggregated and reported within this paper to the extent possible while maintaining privacy from personal health information as required by law. Data are archived within Providence St Joseph Health systems in a HIPAA-secure audited compute environment. For information, contact the Vice President of Information Management at Providence St Joseph Health.
