# Supplementary material for: Risk factors for severe COVID-19 differ by age for hospitalized adults
Source: Sci Rep. 2022 Apr 28;12:6568. doi: 10.1038/s41598-022-10344-3 (PMC9050669; doi:10.1038/s41598-022-10344-3)
Supplement: Supplementary file 1 — Supplementary Information. [file 41598_2022_10344_MOESM1_ESM.docx]

**Supplemental Materials**

for ***Risk factors for severe COVID-19 differ by age for hospitalized adults***


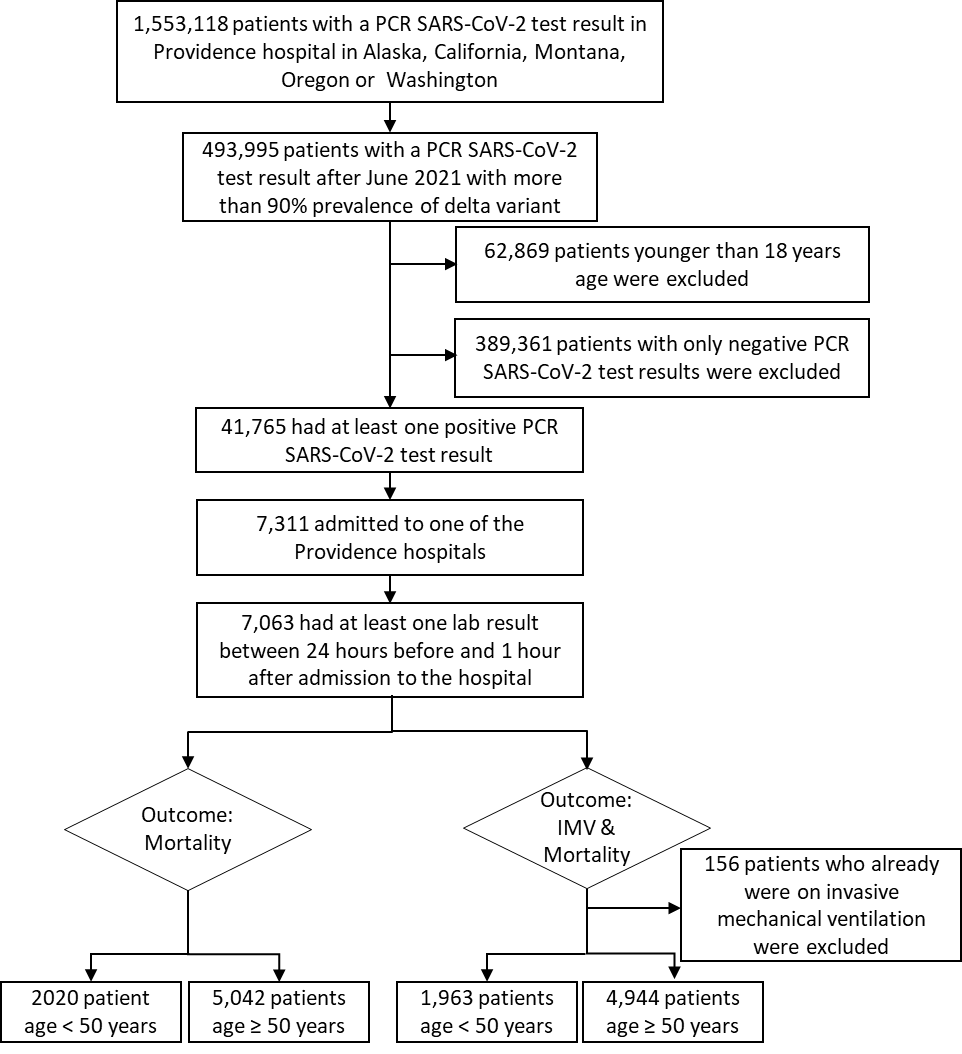


**Supplemental Figure 1:** Cohort selection from PSJH-EHR data.

**
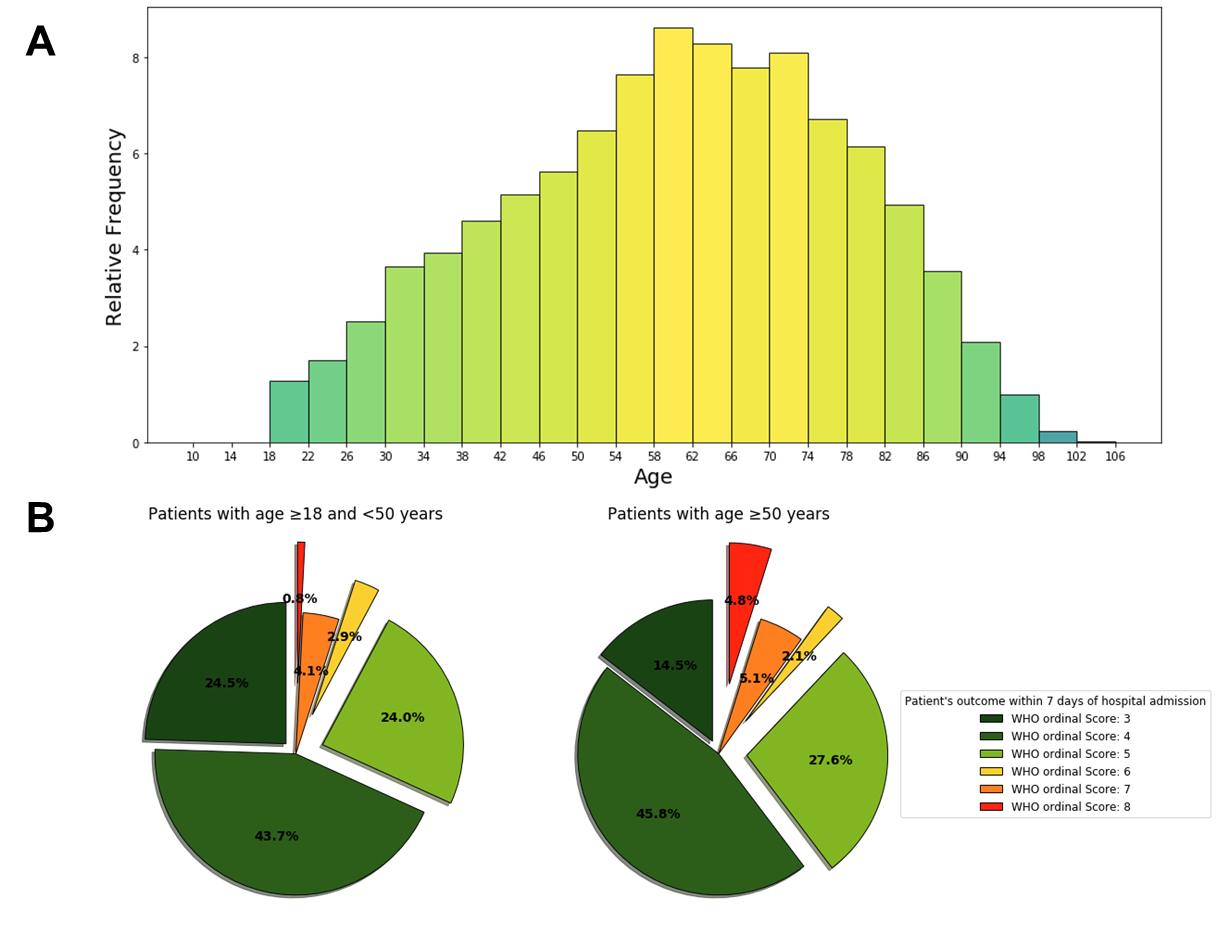
**

**Supplemental Figure 2:** (A) Frequency histogram of hospitalized patients based on their age. (B) Frequency of hospitalized patients’ maximum WHO ordinal score within 7 days of hospitalization based on their age.

**
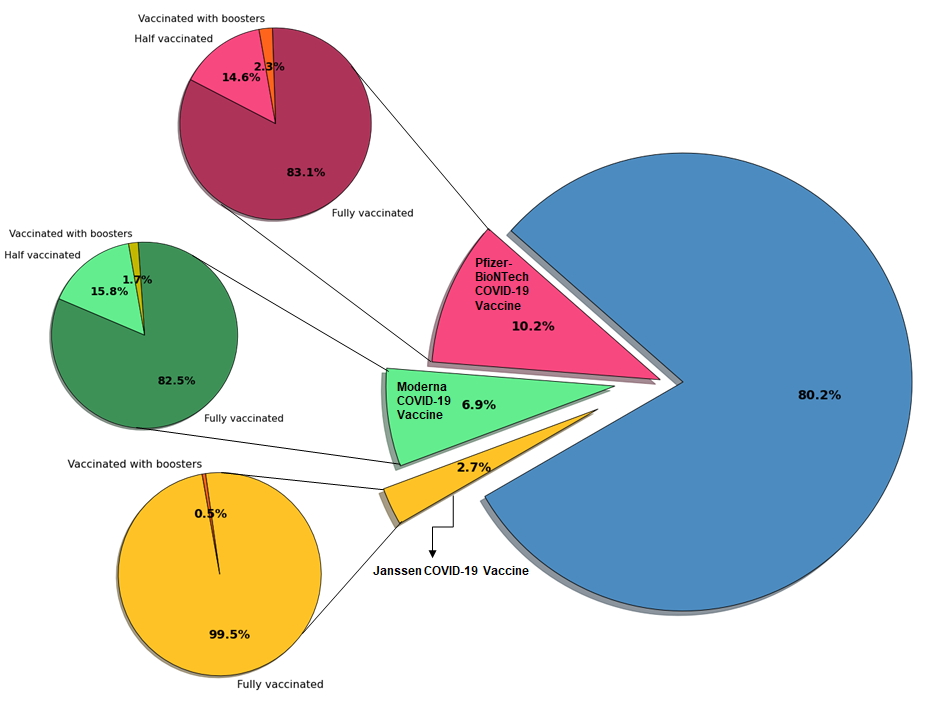
**

**Supplemental Figure 3:** Vaccination status in hospitalized patients


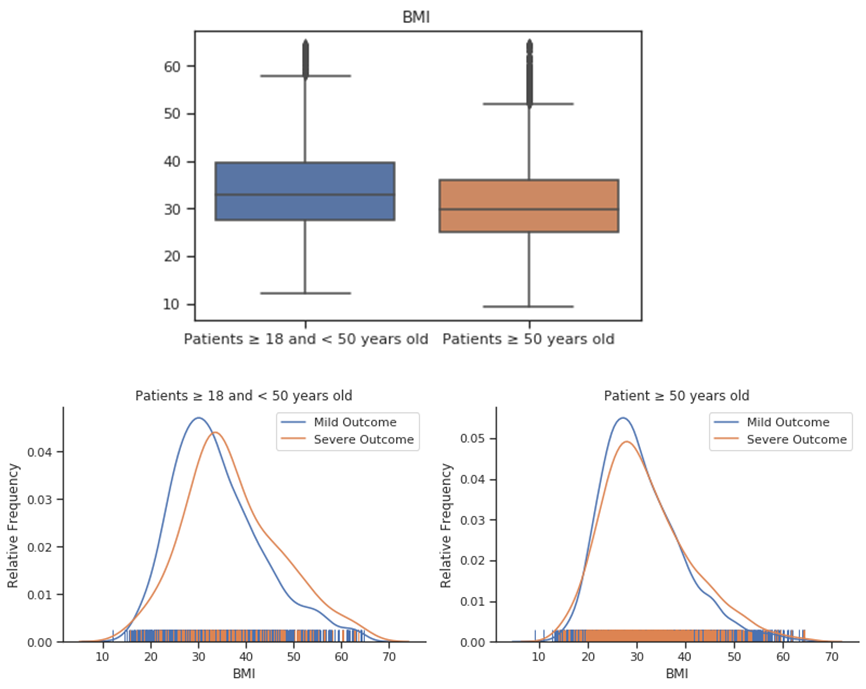


**Supplemental Figure 4:** Distribution of patients’ BMI for invasive mechanical ventilation and mortality outcome in different age groups

**
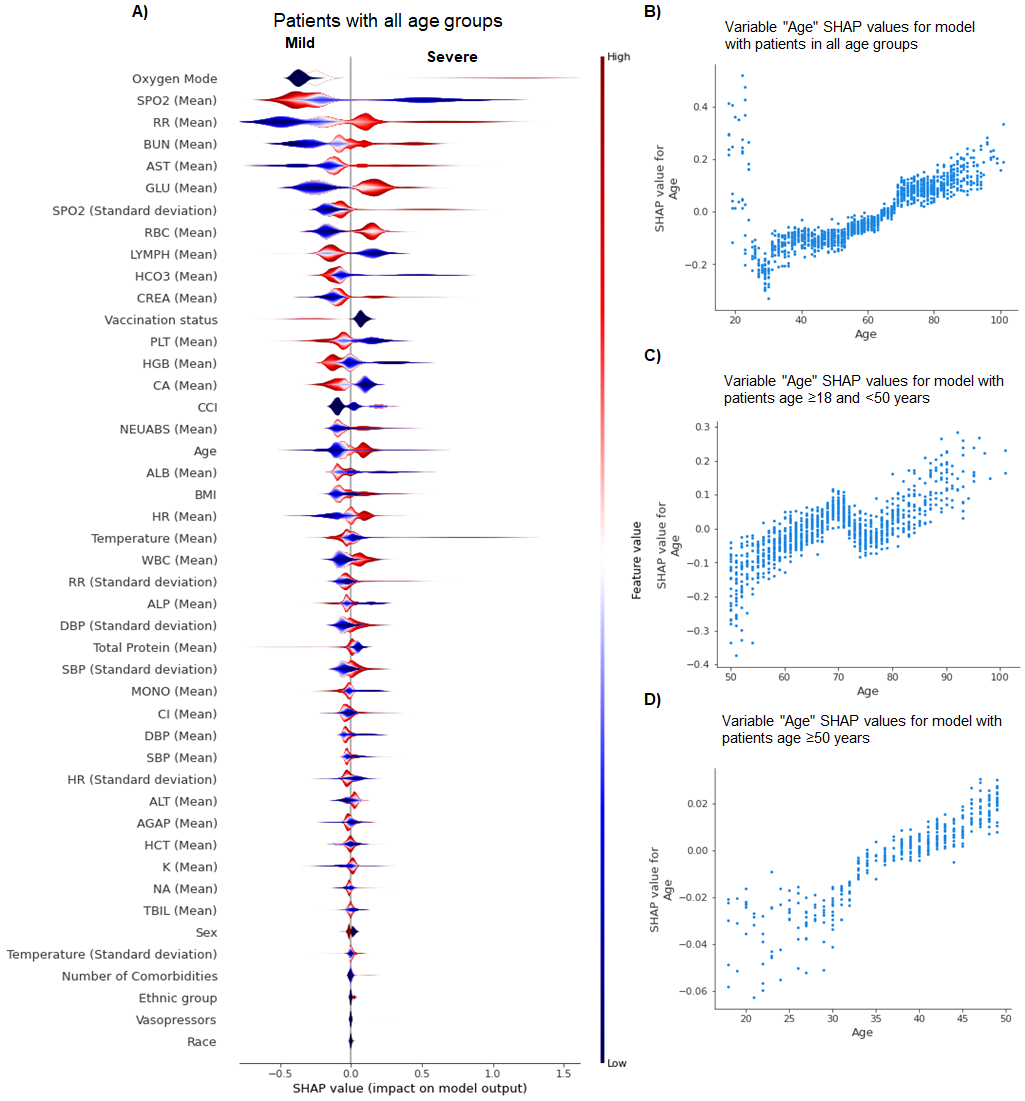
**

**Supplemental Figure 5: Feature importance for all-age model of severe COVID-19 outcomes in hospitalized patients:** A) Gradient Boosting Decision Tree feature importance and the influence of higher and lower values of the risk factors on the all-age group population outcome. Note that the left side of this graph represents reduced risk of the outcomes of severe COVID-19 (critical illness and death) and the right side of the graph represents the increased risk of severe COVID-19. Nominal classes are binary [0, 1]. For sex, female is 0 (blue) and for race, White is 0 (blue), B: Variable “Age” contribution in prediction process of model for patients with all age groups, C: Variable “Age” contribution in prediction process of model for patients’ age ≥ 18 and < 50 years, D: Variable “Age” contribution in prediction process of model for patients’ age ≥ 50 years.


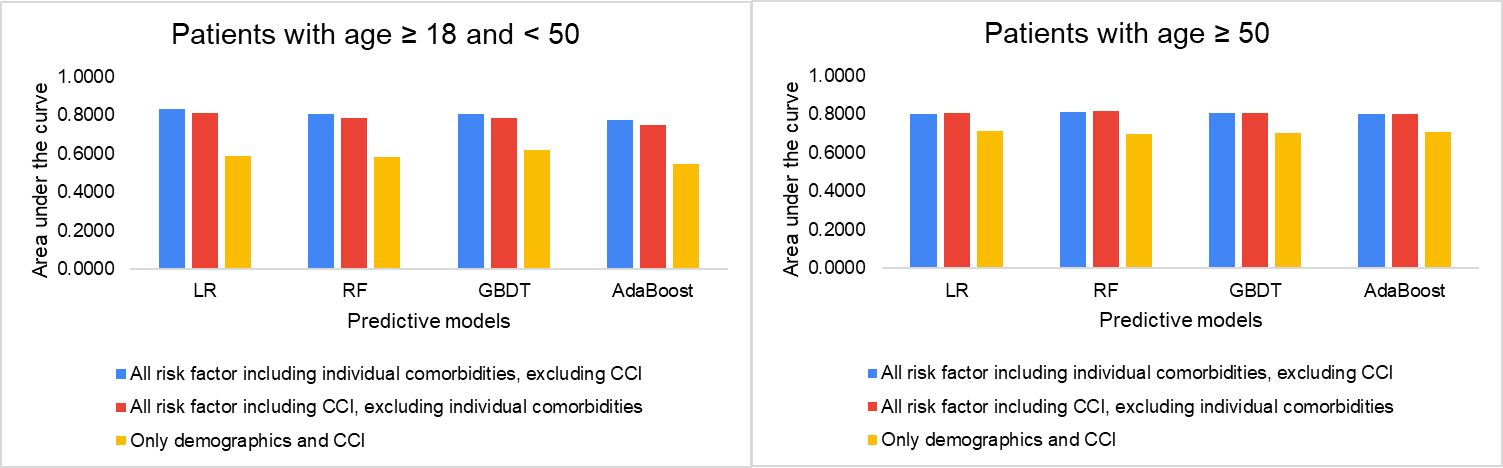


**Supplemental Figure 6: Comparison of area under the ROC curve for predictive models using different sets of risk factors for younger and older subpopulations.**


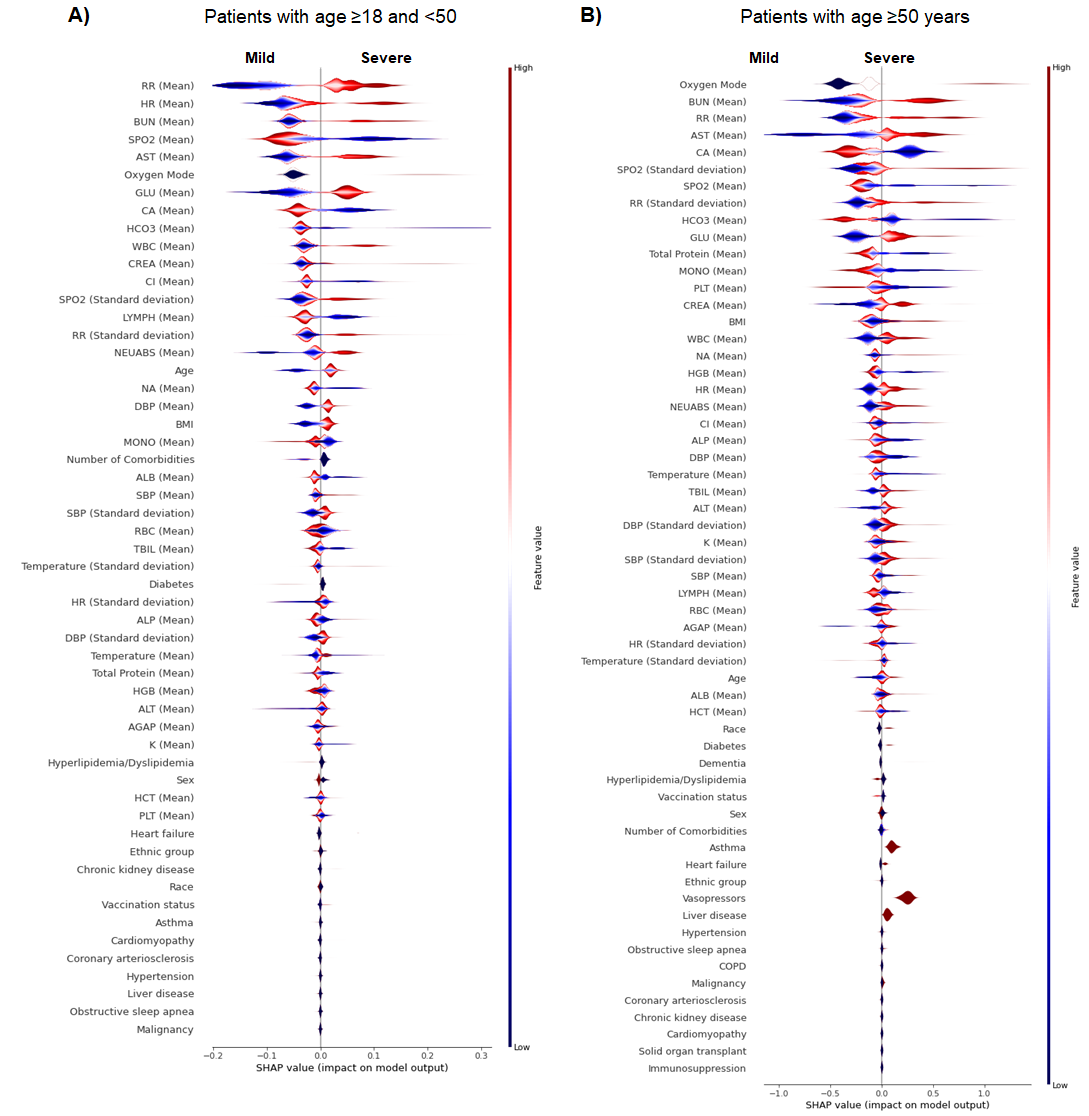


**Supplemental Figure 7: Feature importance for age-stratified models of severe COVID-19 outcomes in hospitalized patients.** These models include individual pre-existing comorbidities, and not the Charlson Comorbidity Index. A) Gradient Boosting Decision Tree feature importance and the influence of higher and lower values of the risk factors on the patient with age ≥ 18 and < 50 years outcome. B) Gradient Boosting Decision Tree feature importance and the influence of higher and lower values of the risk factors on the patient with age ≥ 50 years outcome. Note that the left side of this graph represents reduced risk of the outcomes of severe COVID-19 (critical illness and death) and the right side of the graph represents the increased risk of severe COVID-19. Nominal classes are binary [0, 1]. For sex, female is 0 (blue) and for race, White is 0 (blue).


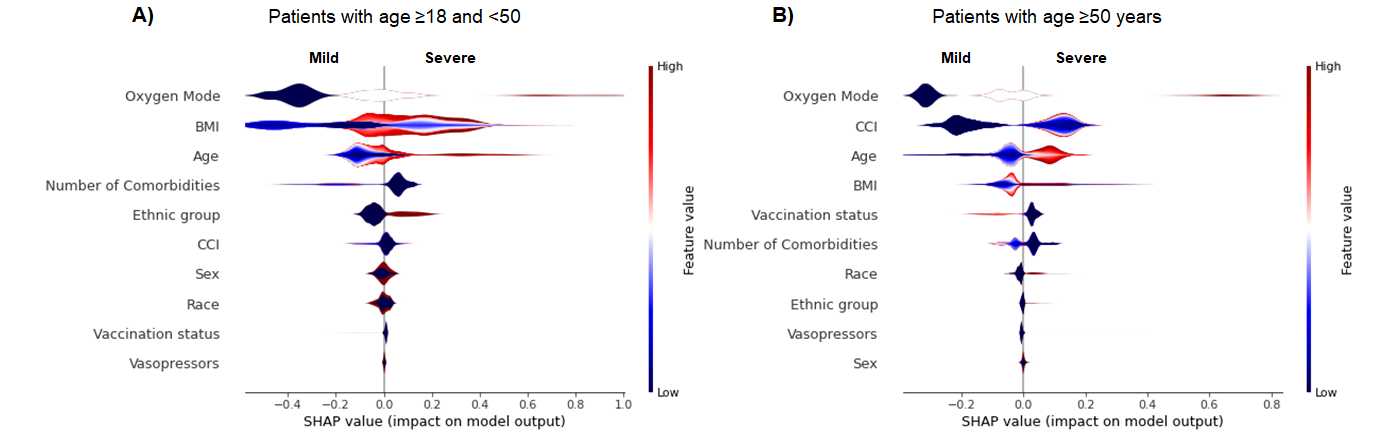


**Supplemental Figure 8: Feature importance for age-stratified models of severe COVID-19 outcomes in hospitalized patients.** These models include only patients’ demographics and Charlson Comorbidity Index. A) Gradient Boosting Decision Tree feature importance and the influence of higher and lower values of the risk factors on the patient with age ≥ 18 and < 50 years outcome. B) Gradient Boosting Decision Tree feature importance and the influence of higher and lower values of the risk factors on the patient with age ≥ 50 years outcome. Note that the left side of this graph represents reduced risk of the outcomes of severe COVID-19 (critical illness and death) and the right side of the graph represents the increased risk of severe COVID-19. Nominal classes are binary [0, 1]. For sex, female is 0 (blue) and for race, White is 0 (blue).

**Supplemental Table 1: Implementation of World Health Organization Ordinal Scale (WOS)**

| **Patient State** | **Descriptor** | **Score** | **Implementation** |
| --- | --- | --- | --- |
| **Uninfected** | No clinical or virological evidence | 0 | Not applicable: all patients in study were hospitalized. |
| **Ambulatory** | No limitation of activities | 1 | Not applicable: all patients in study were hospitalized. |
|  | Limitation of activities | 2 | Not applicable: all patients in study were hospitalized. |
| **Hospitalized**  **mild disease** | Hospitalized, no oxygen therapy | 3 | Encounter |
|  | Oxygen by mask or nasal prongs | 4 | Nursing flowsheets, step function |
|  | Non-invasive ventilation of high-flow oxygen | 5 | Nursing flowsheets, step function |
| **Hospitalized**  **severe disease** | Intubation and mechanical ventilation | 6 | Nursing flowsheets, step function |
|  | Ventilation and additional organ support: pressors, renal replacement therapy, dialysis, extracorporeal membrane oxygenation (ECMO) | 7 | Vasopressor in medication administration records. RxNorm ingredient: 3616 dobutamine, 3628 dopamine, 3966 ephedrine, 3992 epinephrine, 6963 midodrine, 7512 norepinephrine, 8163 phenylephrine, 11149 vasopressin (USP)  Renal replacement therapy: Nursing flowsheets, step function  ECMO: Nursing flowsheets, step function |
|  | Death | 8 | Date of death is recorded |

**Supplemental Table 2: Terms, SNOMED-CT parent codes and ICD-10 codes**

| **Term** | **SNOMED parent codes*** | **ICD10 codes** |
| --- | --- | --- |
| **Hypertension** | 31992008, 59621000 | I12.0, I12.9, I15.8, I15.9, I10, I11.0, I11.9, I13, I13.0, I13.1, I13.2, I13.9, I15, I15.0, I15.1, I15.2, I15.8, I15.9 |
| **Coronary Arteriosclerosis** | 53741008, 443502000 | I25.10, I25.110, I25.111, I25.118, I25.119, I25.708, I25.709, I25.718, I25.719, I25.728, I25.738, I25.750, I25.751, I25.758, I25.759, I25.799, I25.811, I25.812, I25.10, I25.111, I25.119, I25.759, I25.810, I25.811, Q24.5 |
| **Heart failure** | 84114007 | I09.81, I11.0, I11.9, I13.0, I13.10, I13.11, I13.2, I50.20, I50.21, I50.22, I50.23, I50.30, I50.31, I50.32, I50.33, I50.40, I50.41, I50.42, I50.43, I50.810, I50.811, I50.812, I50.813, I50.814, I50.82, I50.83, I50.84, I50.89, I50.9, I97.130, I97.131 |
| **Cardiomyopathy** | 85898001 | A36.81, B33.24, I25.5, I42.0, I42.1, I42.2, I42.5, I42.6, I42.7, I42.9, I43, O90.3 |
| **Diabetes mellitus (Includes Type 1 and Type 2)** | 46635009,44054006 | E10.x, E11.x, E12.x, E13.0, E13.x, E14.x |
| **Chronic obstructive pulmonary disease (COPD)** | 13645005 | J43.9, J44.0, J44.1, J44.9 |
| **Asthma** | 195967001 | J45.20, J45.21, J45.22, J45.30, J45.31, J45.32, J45.40, J45.41, J45.42, J45.50, J45.51, J45.52, J45.901, J45.902, J45.909,  J45.998 |
| **Obstructive sleep apnea** | 78275009 | G47.30, G47.31, G47.33, G47.37, P28.3 |
| **Chronic kidney disease** | 709044004 | D63.1, E08.22, E08.65, E09.22, E10.22, E11.22, E11.65, I12.0, I12.9, I13.0, I13.10, I13.2, N03.9, N18.1, N18.2, N18.3, N18.4, N18.5, N18.9 |
| **Liver disease** | 235856003 | B18.x, K70.0–K70.3, K70.9, K71.3–K71.5, K71.7, K73.x, K74.x, K76.0, K76.2–K76.4, K76.8, K76.9, Z94.4, I85.0, I85.9, I86.4, I98.2, K70.4, K71.1, K72.1, K72.9, K76.5, K76.6, K76.7 |
| **Malignant neoplastic disease, current or past** | 363346000 | C00.x–C26.x, C30.x–C34.x, C37.x–C41.x, C43.x, C45.x–C58.x, C60.x–C76.x, C81.x–C85.x, C88.x, C90.x–C97.x, C77.x–C80.x |
| **Dyslipidemia, Hyperlipidemia** | 370992007, 55822004 | E78.4, E78.5, E78.3, E78.4, E78.5 |
| **History of solid organ transplant (includes heart, lungs, kidney, liver)** | 739025007,737295003, 737296002, 737297006 | T86.10, T86.11, T86.12, T86.13, T86.19, Z94.0, T86.30, T86.31, T86.32, T86.810, T86.811, T86.812, T86.818, T86.819, Z48.24, Z48.280, Z94.2, Z94.3, T86.40, T86.41, T86.43, T86.49, Z48.23, Z94.4, I25.750, I25.751, I25.758, I25.759, I25.761, I25.768, I25.769, I25.811, I25.812, T86.20, T86.21, T86.22, T86.298, T86.30, T86.31, T86.32, Z48.21, Z48.280, Z94.1, Z94.3 |
| **Immunosuppression** | 737300001, 370388006, 370391006, 234532001, 86406008, 62479008 | D81.9, D83.0, D83.1, D83.9, D82.9, D82.8, D82.3, D80.5, D80.9, D82.2, D84.9, D81.1, D81.2, D81.0, Z94.81, B20, O98.711, O98.712, O98.713, O98.719, O98.72, Z21 |
| **Dementia (all causes)** | 52448006 | F00.x–F03.x, F05.1, G30.x, G31.1 |

*Terms were defined as the parent code and all children under that parent.

**Supplemental Table 3: Mean and standard deviation values and missing percentage of laboratory results among hospital patients with COVID-19 by severity**

| **Variable** | **Patients with age ≥ 18 and < 50 years**  **(n=1,963)** | | |  | **Patients with age ≥ 50 years**  **(n=4,944)** | | |  |
| --- | --- | --- | --- | --- | --- | --- | --- | --- |
|  | **Less Severe** | **Severe** | **P-value** | **Missing (%)** | **Less Severe** | **Severe** | **P-value** | **Missing (%)** |
| **RBC (std),** x10^12^/L | 4.732 (0.714) | 4.828 (0.753) | 0.017 | 1.375 | 4.505 (0.764) | 4.524 (0.845) | 0.282 | 2.306 |
| **WBC (std) ,** x10^9^/L | 8.360 (8.424) | 8.987 (5.479) | 0.043 | 1.375 | 8.236 (6.261) | 9.619 (8.207) | <0.001 | 2.286 |
| **Neutrophils (std),** x10^9^/L | 6.213 (3.822) | 7.381 (4.739) | 0.014 | 8.507 | 6.279 (3.968) | 7.439 (4.596) | <0.001 | 6.271 |
| **Lymphocytes (std) ,** x10^9^/L | 1.259 (6.105) | 0.968 (0.669) | <0.001 | 8.660 | 1.153 (4.351) | 1.164 (4.595) | <0.001 | 6.373 |
| **Eosinophils (std),** x10^9^/L | 0.046 (0.111) | 0.019 (0.065) | <0.001 | 29.699 | 0.050 (0.144) | 0.024 (0.069) | <0.001 | 25.571 |
| **Basophils (std),** x10^9^/L | 0.020 (0.032) | 0.018 (0.034) | 0.128 | 26.999 | 0.021 (0.089) | 0.019 (0.033) | 0.142 | 24.074 |
| **Platelets (std),** x10^9^/L | 238.745 (99.499) | 227.856 (95.169) | 0.127 | 1.528 | 224.315 (102.507) | 213.450 (101.504) | <0.001 | 2.448 |
| **Hematocrit (std),** %  **Male**  **Female** | 43.125 (5.569)  38.315 (5.424) | 44.052 (5.462)  37.604 (5.928) | 0.342  0.278 | 1.324 | 41.088 (6.533)  38.935 (5.667) | 41.565 (7.143)  38.206 (6.669) | 0.243  0.198 | 2.246 |
| **Hemoglobin (std),** g/dL  **Male**  **Female** | 14.588 (1.989)  12.659 (1.956) | 14.978 (1.952)  12.389 (2.084) | 0.025  0.020 | 1.273 | 13.789 (2.340)  12.846 (1.993) | 13.902 (2.530)  12.484 (2.279) | 0.175  0.143 | 2.165 |
| **Globulin (std),** g/dL | 3.709 (0.842) | 3.787 (0.893) | 0.103 | 21.599 | 3.632 (0.845) | 3.696 (0.892) | 0.468 | 21.080 |
| **Sodium (std),** mmol/L | 135.828 (4.100) | 134.966 (4.840) | <0.001 | 7.030 | 135.588 (4.855) | 135.746 (6.133) | 0.213 | 2.691 |
| **Potassium (std),** mmol/L | 3.865 (0.545) | 3.895 (0.635) | 0.280 | 7.132 | 3.999 (0.597) | 4.125 (0.663) | <0.001 | 2.650 |
| **Calcium (std),** mmol/L | 8.749 (0.613) | 8.453 (0.615) | <0.001 | 7.132 | 8.780 (0.618) | 8.592 (0.644) | <0.001 | 2.792 |
| **Magnesium (std),** mg/dL | 2.052 (0.495) | 1.970 (0.450) | <0.001 | 84.921 | 1.975 (0.353) | 2.123 (0.609) | <0.001 | 82.986 |
| **Albumin (std),** g/dL | 3.652 (0.704) | 3.428 (0.738) | <0.001 | 11.105 | 3.474 (0.659) | 3.261 (0.732) | <0.001 | 8.557 |
| **Creatinine (std),** mg/dL | 1.093 (1.339) | 1.300 (1.349) | <0.001 | 6.928 | 1.370 (1.509) | 1.642 (1.325) | <0.001 | 2.630 |
| **AST (std),** U/L | 79.670 (141.354) | 104.125 (107.617) | <0.001 | 11.563 | 58.203 (65.267) | 107.067 (250.009) | <0.001 | 9.104 |
| **ALT (std),** U/L | 71.945 (97.304) | 72.832 (60.649) | 0.362 | 11.462 | 47.197 (59.073) | 67.058 (118.011) | <0.001 | 9.549 |
| **LDH (std),** U/L | 462.544 (220.515) | 579.333 (281.813) | <0.001 | 79.317 | 401.303 (184.575) | 541.781 (370.662) | <0.001 | 83.512 |
| **Glucose (std),** mg/dL | 146.594 (93.950) | 159.092 (95.668) | <0.001 | 6.317 | 147.074 (72.026) | 172.005 (99.159) | <0.001 | 1.881 |
| **Bilirubin (std),** mg/dL | 0.685 (0.834) | 0.801 (1.812) | 0.128 | 11.768 | 0.702 (0.543) | 0.794 (0.605) | <0.001 | 9.346 |
| **Anion Gap (std),** mmol/L | 10.157 (4.175) | 11.196 (4.199) | 0.023 | 7.590 | 9.702 (3.594) | 11.421 (4.457) | <0.001 | 3.054 |
| **Alkaline (std),** U/L | 94.852 (77.846) | 92.745 (64.137) | <0.001 | 11.309 | 94.434 (71.613) | 94.657 (64.072) | 0.447 | 8.861 |
| **Monocytes (std),** x10^9^/L | 0.519 (0.385) | 0.457 (0.454) | <0.001 | 8.660 | 0.592 (0.452) | 0.603 (1.197) | <0.001 | 6.453 |
| **BUN (std),** mg\dL | 14.835 (13.570) | 18.895 (14.074) | <0.001 | 9.119 | 23.897 (17.242) | 34.765 (22.949) | <0.001 | 2.954 |
| **Chloride (std),** mmol/L | 101.034 (5.045) | 100.268 (5.728) | <0.001 | 7.030 | 100.944 (5.549) | 101.350 (6.635) | 0.455 | 2.670 |
| **Bicarbonate (std),** mmol/L | 24.907 (4.236) | 23.899 (4.750) | <0.001 | 7.285 | 25.205 (3.931) | 23.234 (4.956) | <0.001 | 2.610 |
| **Ferritin (std),** ng/mL | 1169.543 (1667.133) | 1398.247 (1524.964) | <0.001 | 80.489 | 1001.510 (1108.519) | 1160.807 (1155.168) | <0.001 | 83.107 |
| **Procalcitonin (std).** ng/mL | 2.595 (25.863) | 4.042 (19.127) | <0.001 | 65.919 | 1.455 (6.573) | 3.483 (13.963) | <0.001 | 83.107 |
| **Total Protein (std),** g/dL | 7.348 (0.740) | 7.196 (0.795) | <0.001 | 11.309 | 7.098 (0.730) | 6.939 (0.827) | <0.001 | 8.861 |
| **C-reactive (std)**, mg/L | 35.945 (51.749) | 36.623 (56.429) | <0.001 | 64.085 | 37.197 (58.468) | 46.909 (75.217) | <0.001 | 69.350 |
| **Prothrombin (std),** sec | 13.933 (2.971) | 14.486 (2.844) | <0.001 | 77.331 | 15.694 (8.245) | 16.840 (7.262) | <0.001 | 72.385 |
| **BUN/ Creat (std),** ratio | 14.596 (6.313) | 14.896 (5.605) | 0.343 | 25.420 | 19.234 (7.766) | 23.255 (11.151) | <0.001 | 24.924 |
| **INR (std),** ratio | 1.150 (0.318) | 1.188 (0.295) | <0.001 | 77.331 | 1.324 (0.869) | 1.432 (0.756) | <0.001 | 72.122 |
| **D-Dimer (std),**  µg/ml | 1.312 (2.586) | 2.602 (6.056) | <0.001 | 63.984 | 1.667 (2.849) | 3.461 (10.168) | <0.001 | 69.876 |

**Supplemental Table 4: Vital sign data among patients with COVID-19 according to the severity**

| **Variable** | **Patients ≥ 18 and < 50 years**  **(n=706)** | | | **Patients >= 50 years old**  **(n=2531)** | | |
| --- | --- | --- | --- | --- | --- | --- |
|  | Less Severe | Severe | P-value | Less Severe | Severe | P-value |
| **SBP** | 124.848 (17.381) | 125.949 (18.416) | 0.143 | 130.859 (20.502) | 128.440 (22.401) | <0.001 |
| **DBP** | 75.321 (11.774) | 76.437 (12.250) | 0.130 | 73.193 (12.219) | 71.963 (12.723) | <0.001 |
| **RR** | 22.422 (5.631) | 27.148 (8.235) | <0.001 | 21.747 (4.708) | 25.363 (5.960) | <0.001 |
| **SpO_2_** | 94.341 (3.441) | 91.086 (5.162) | <0.001 | 93.821 (3.408) | 91.751 (5.021) | <0.001 |
| **HR** | 97.374 (16.404) | 106.390 (16.577) | <0.001 | 88.989 (16.821) | 94.054 (19.447) | <0.001 |
| **Body Temp.** | 99.178 (1.652) | 99.432 (1.594) | 0.013 | 98.839 (1.678) | 98.863 (1.613) | 0.080 |

**Supplemental Table 5: Prediction performance and 95% confidence interval on the test set for models with full set of features.**

| **Age group** | **Classifier** | **AUROC** | **PR-AUC** | **TPR** | **TNR** | **PPV** | **NPV** | **LKR+** | **LKR-** |
| --- | --- | --- | --- | --- | --- | --- | --- | --- | --- |
| **Generalized model for patients in all age groups** | LR | 0.7937 (0.75-0.83) | 0.5135 (0.44-0.59) | 0.7225 (0.65-0.79) | 0.7223 (0.70-0.75) | 0.2700 (0.23-0.31) | 0.9482 (0.94-0.96) | 2.6016 (2.29-2.96) | 0.3841 (0.30-0.49) |
|  | RF | 0.7967 (0.76-0.84) | 0.5132 (0.44-0.59) | 0.7225 (0.66-0.79) | 0.7214 (0.70-0.75) | 0.2694 (0.23-0.31) | 0.9482 (0.94-0.96) | 2.5939 (2.28-2.95) | 0.3848 (0.30-0.49) |
|  | GBDT | 0.7974 (0.76-0.84) | 0.5195 (0.45-0.59) | 0.7283 (0.66-0.79) | 0.7305 (0.71-0.76) | 0.2775 (0.24-0.32) | 0.9498 (0.94-0.96) | 2.7023 (2.37-3.08) | 0.3719 (0.29-0.47) |
|  | AdaBoost | 0.7884 (0.75-0.83) | 0.5135 (0.44-0.59) | 0.7225 (0.65-0.79) | 0.7223 (0.70-0.75) | 0.2700 (0.23-0.31) | 0.9482 (0.94-0.96) | 2.6016 (2.29-2.96) | 0.3841 (0.30-0.49) |
| **Age-stratified**  **model for patients**  **age ≥ 18 and < 50 years** | LR | 0.8118 (0.71-0.97) | 0.4752 (0.29-0.66) | 0.7498 (0.59,009) | 0.7432 (0.70,0.79) | 0.1826 (0.11,0.25) | 0.9749 (0.96,0.99) | 2.9202 (2.22,3.85) | 0.3364 (0.18,0.64) |
|  | RF | 0.7855 (0.68-0.89) | 0.4776 (0.29-0.66) | 0.7498 (0.60,0.91) | 0.7514 (0.71,0.80) | 0.1875 (0.12,0.26) | 0.9752 (0.96,0.99) | 3.0165 (2.28,3.98) | 0.3327 (0.17,0.63) |
|  | GBDT | 0.7842 (0.68-0.89) | 0.4744 (0.29-0.66) | 0.7498 (0.59,0.91) | 0.7404 (0.70,0.78) | 0.1810 (0.11,0.25) | 0.9748 (0.96,0.99) | 2.8895 (2.19,3.80) | 0.3376 (0.18,0.64) |
|  | AdaBoost | 0.7517 (0.64-0.86) | 0.4201 (0.24-0.60) | 0.6786 (0.51,0.85) | 0.6776 (0.63,0.73) | 0.1387 (0.08,0.20) | 0.9650 (0.94,0.99) | 2.1047 (1.57,2.83) | 0.4744 (0.28,0.82) |
| **Age-stratified model for patients age ≥ 50 years** | LR | 0.8060 (0.76-0.85) | 0.5088 (0.42-0.60) | 0.7190 (0.64,0.80) | 0.7234 (0.70,0.75) | 0.2644 (0.22,0.31) | 0.9490 (0.93,0.97) | 2.5997 (2.23,3.03) | 0.3884 (0.29,0.52) |
|  | RF | 0.8160 (0.77-0.86) | 0.5393 (0.45-0.63) | 0.7521 (0.68,0.83) | 0.7531 (0.72,0.78) | 0.2964 (0.25,0.35) | 0.9565 (0.94,0.97) | 3.0466 (2.61,3.56) | 0.3292 (0.24,0.45) |
|  | GBDT | 0.8073 (0.76-0.86) | 0.5156 (0.43-0.60) | 0.7273 (0.65,0.81) | 0.7291 (0.70,0.76) | 0.2708 (0.22,0.32) | 0.9508 (0.93,0.97) | 2.6851 (2.30,3.13) | 0.3740 (0.28,0.50) |
|  | AdaBoost | 0.8009 (0.75-0.85) | 0.5148 (0.43-0.60) | 0.7273 (0.65,0.81) | 0.7269 (0.70,0.76) | 0.2691 (0.22,0.32) | 0.9507 (0.93,0.97) | 2.6626 (2.28,3.10) | 0.3752 (0.28,0.50) |

AUROC = area under the receiver-operating characteristic curve. PR-AUC: area under the precision-recall curve. TPR = true positive rate. TNR = true negative rate. LKR+ = positive likelihood ratio. LKR- = negative likelihood ratio. PPV = positive predictive value. NPV = negative predictive value.

**Supplemental Table 6: STROBE Statement -- Checklist of items that should be included in reports of cohort studies**

|  |  | **Item No** | **Recommendation** |
| --- | --- | --- | --- |
| **Yes** | **Title and abstract** | 1 | (*a*) Indicate the study’s design with a commonly used term in the title or the abstract |
|  |  |  | (*b*) Provide in the abstract an informative and balanced summary of what was done and what was found |
|  | **Introduction** | | |
| Yes | Background / rationale | 2 | Explain the scientific background and rationale for the investigation being reported |
| Yes | Objectives | 3 | State specific objectives, including any prespecified hypotheses |
|  | **Methods** | | |
| Yes | Study design | 4 | Present key elements of study design early in the paper |
| Yes | Setting | 5 | Describe the setting, locations, and relevant dates, including periods of recruitment, exposure, follow-up, and data collection |
| Yes | Participants | 6 | (*a*) Give the eligibility criteria, and the sources and methods of selection of participants. Describe methods of follow-up |
|  |  |  | (*b*) For matched studies, give matching criteria and number of exposed and unexposed |
| Yes | Variables | 7 | Clearly define all outcomes, exposures, predictors, potential confounders, and effect modifiers. Give diagnostic criteria, if applicable |
| Yes | Data sources / measurement | 8* | For each variable of interest, give sources of data and details of methods of assessment (measurement). Describe comparability of assessment methods if there is more than one group |
| N/A | Bias | 9 | Describe any efforts to address potential sources of bias |
| Yes | Study size | 10 | Explain how the study size was arrived at |
| Yes | Quantitative variables | 11 | Explain how quantitative variables were handled in the analyses. If applicable, describe which groupings were chosen and why |
| Yes | Statistical methods | 12 | (*a*) Describe all statistical methods, including those used to control for confounding |
|  |  |  | (*b*) Describe any methods used to examine subgroups and interactions |
|  |  |  | (*c*) Explain how missing data were addressed |
|  |  |  | (*d*) If applicable, explain how loss to follow-up was addressed |
|  |  |  | (*e*) Describe any sensitivity analyses |
|  | **Results** | | |
| Yes | Participants | 13* | (a) Report numbers of individuals at each stage of study—eg numbers potentially eligible, examined for eligibility, confirmed eligible, included in the study, completing follow-up, and analysed |
|  |  |  | (b) Give reasons for non-participation at each stage |
|  |  |  | (c) Consider use of a flow diagram |
| Yes | Descriptive data | 14* | (a) Give characteristics of study participants (eg demographic, clinical, social) and information on exposures and potential confounders |
|  |  |  | (b) Indicate number of participants with missing data for each variable of interest |
|  |  |  | (c) Summarise follow-up time (eg, average and total amount) |
| Yes | Outcome data | 15* | Report numbers of outcome events or summary measures over time |
| Yes | Main results | 16 | (*a*) Give unadjusted estimates and, if applicable, confounder-adjusted estimates and their precision (eg, 95% confidence interval). Make clear which confounders were adjusted for and why they were included |
|  |  |  | (*b*) Report category boundaries when continuous variables were categorized |
|  |  |  | (*c*) If relevant, consider translating estimates of relative risk into absolute risk for a meaningful time period |
| N/A | Other analyses | 17 | Report other analyses done—eg analyses of subgroups and interactions, and sensitivity analyses |
|  | **Discussion** | | |
| Yes | Key results | 18 | Summarize key results with reference to study objectives |
| Yes | Limitations | 19 | Discuss limitations of the study, taking into account sources of potential bias or imprecision. Discuss both direction and magnitude of any potential bias |
| Yes | Interpretation | 20 | Give a cautious overall interpretation of results considering objectives, limitations, multiplicity of analyses, results from similar studies, and other relevant evidence |
| Yes | Generalisability | 21 | Discuss the generalisability (external validity) of the study results |
|  | **Other information** | | |
| Yes | Funding | 22 | Give the source of funding and the role of the funders for the present study and, if applicable, for the original study on which the present article is based |

*Give information separately for exposed and unexposed groups.
